# Supplementary figures and images for: Sores of boreal moose reveal a previously unknown genetic lineage of parasitic nematode within the genus Onchocerca
Source: PLoS One. 2023 Jan 11;18(1):e0278886. doi: 10.1371/journal.pone.0278886 (PMC9833588; doi:10.1371/journal.pone.0278886)

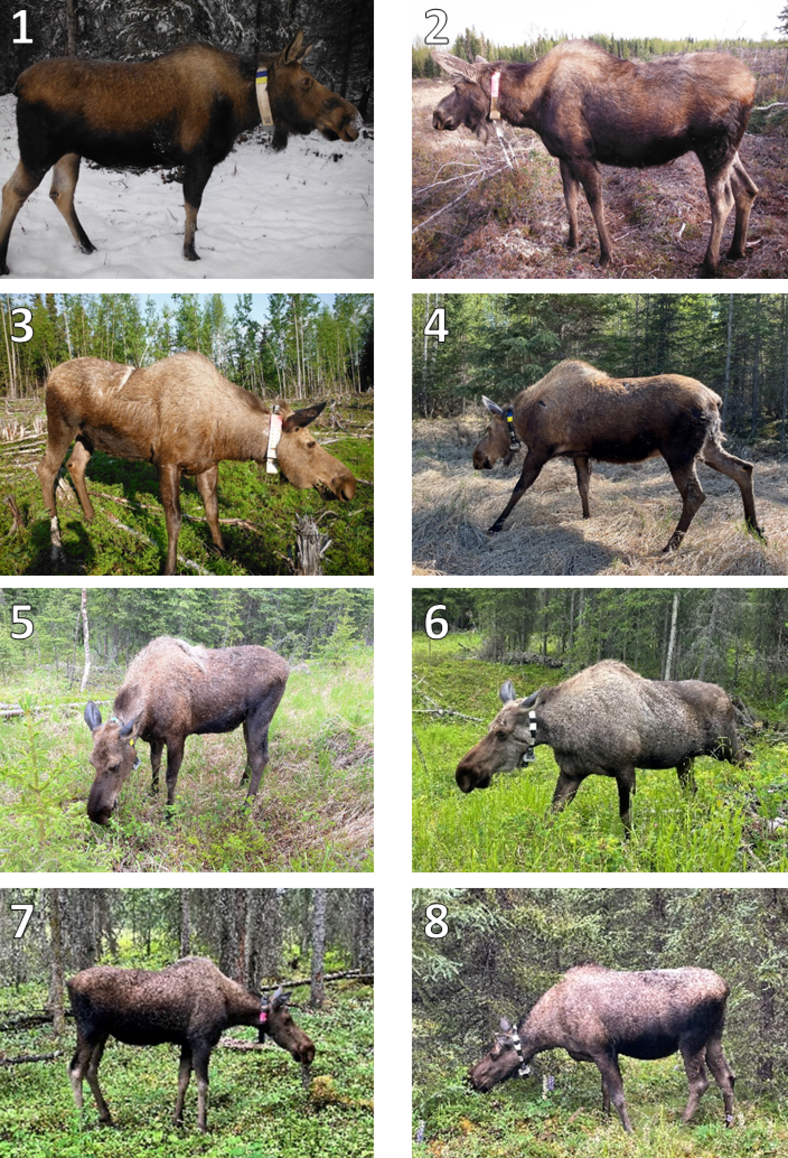

Supplement: S1 Fig — Photographs showing the eight stages of molt scores. Numbers correlated to molt scores in Table 1. (TIF) [file pone.0278886.s001.tif]

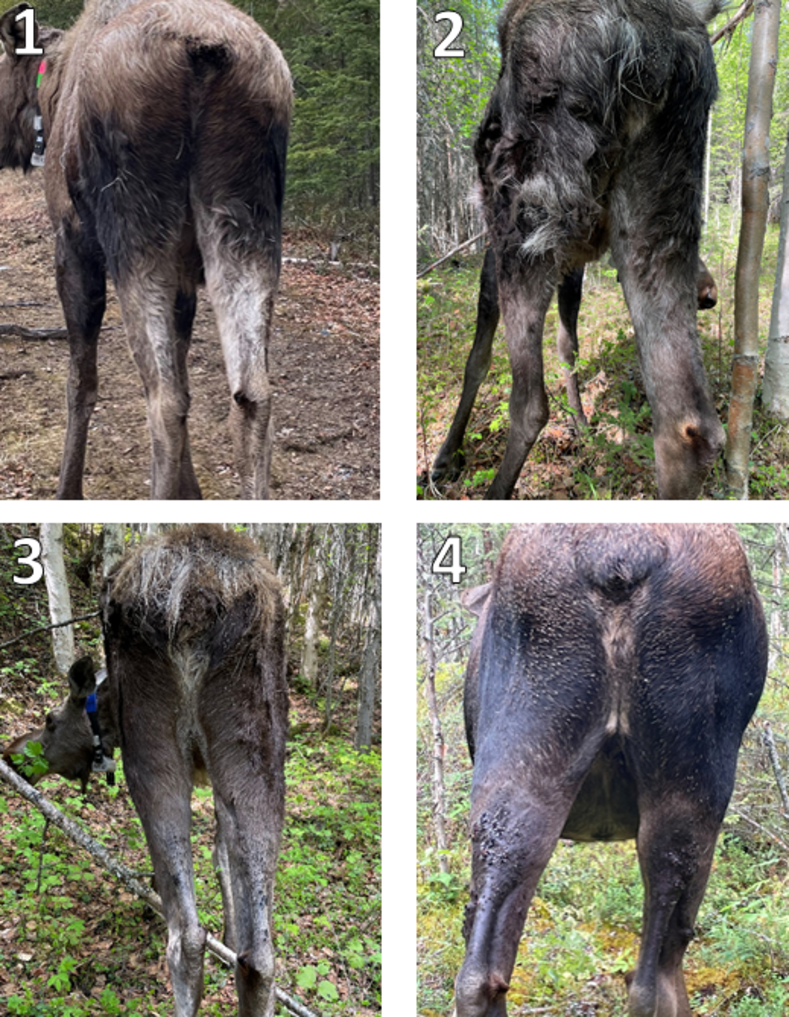

Supplement: S2 Fig — Photographs showing the four stages of hock scores. Numbers correlated to hock scores in Table 1. (TIF) [file pone.0278886.s002.tif]
