# Supplementary material for: Sores of boreal moose reveal a previously unknown genetic lineage of parasitic nematode within the genus Onchocerca
Source: PLoS One. 2023 Jan 11;18(1):e0278886. doi: 10.1371/journal.pone.0278886 (PMC9833588; doi:10.1371/journal.pone.0278886)
Supplement: Attachment — Submitted filename: Response to Reviewers.docx [file pone.0278886.s004.docx]

Manuscript PONE-D-22-25378R1

Sores of boreal moose reveal a previously unknown genetic lineage of parasitic nematode within the genus *Onchocerca*

**Answers to the editor and the reviewers**

We are thankful for the editor and reviewers’ constructive comments and attention to detail that helped to improve and clarify the manuscript. In the following we address the comments individually. We also highlighted the changes in the manuscript by track changes.

**Journal Requirements**

Please review your reference list to ensure that it is complete and correct. If you have cited papers that have been retracted, please include the rationale for doing so in the manuscript text, or remove these references and replace them with relevant current references. Any changes to the reference list should be mentioned in the rebuttal letter that accompanies your revised manuscript. If you need to cite a retracted article, indicate the article’s retracted status in the References list and also include a citation and full reference for the retraction notice.

**Response:** All citations have been check for accuracy and none have been retracted.

**Reviewers' comments**

**Reviewer #2:** Benedict et al. sores of boreal moose....

The authors have drastically improved the manuscript. I have a few minor suggestions below to help improve clarity of some sentences. Nice study with interesting findings.

**Response:** Thank you for your help and guidance! We appreciate your attention to detail in catching these typos and grammatical error, and have corrected them accordingly.

Track changed version; 114-116; new sentence isn’t clear to me. Perhaps it’s the use of ‘what’ in line 115?
**Response:** The typo has been corrected; ‘what’ has been changed to ‘that’ (line 106).

Track changed version line 137; can thermal imagery capture inflammation? Perhaps ‘ to track hair loss as a possible indicator of inflammation’?

**Response:** It would be hard to decipher warming due to just loss of hair versus warming due to inflammation; ‘and inflammation’ has been removed (lines 117-118).

Track changed version line 204-207; do these sentences say the same thing?

**Response:** Yes, the sentences have been combined (lines 174-176).

Track changed version line 338; need complete sentence after semicolon

**Response:** The sentence has been made complete (lines 293-294).
